# Modulation of P2X7 Receptor during Inflammation in Multiple Sclerosis

**DOI:** 10.3389/fimmu.2017.01529

**Published:** 2017-11-15

**Authors:** Susanna Amadio, Chiara Parisi, Eleonora Piras, Paola Fabbrizio, Savina Apolloni, Cinzia Montilli, Sabina Luchetti, Serena Ruggieri, Claudio Gasperini, Franco Laghi-Pasini, Luca Battistini, Cinzia Volonté

**Affiliations:** ^1^Cellular Neurobiology Unit, Santa Lucia Foundation, Rome, Italy; ^2^Institute of Cell Biology and Neurobiology, Consiglio Nazionale Delle Ricerche (CNR), Rome, Italy; ^3^Neuroimmunology Unit, Santa Lucia Foundation, Rome, Italy; ^4^Department of Medical Sciences, Surgery and Neurosciences, University of Siena, Siena, Italy; ^5^Neuroimmunology Research Group, Netherlands Institute for Neuroscience, Amsterdam, Netherlands; ^6^Neurology Unit “Lancisi”, San Camillo Forlanini Hospital, Rome, Italy; ^7^Department of Neurology and Psychiatry, University of Rome “Sapienza”, Rome, Italy

**Keywords:** astrocytes, monocyte chemoattractant protein-1, monocytes, multiple sclerosis, neuroinflammation, P2X7 receptor

## Abstract

Multiple sclerosis (MS) is characterized by macrophage accumulation and inflammatory infiltrates into the CNS contributing to demyelination. Because purinergic P2X7 receptor (P2X7R) is known to be abundantly expressed on cells of the hematopoietic lineage and of the nervous system, we further investigated its phenotypic expression in MS and experimental autoimmune encephalomyelitis conditions. By quantitative reverse transcription polymerase chain reaction and flow cytometry, we analyzed the P2X7R expression in human mononuclear cells of peripheral blood from stable and acute relapsing-remitting MS phases. Human monocytes were also challenged *in vitro* with pro-inflammatory stimuli such as the lipopolysaccharide, or the P2X7R preferential agonist 2′(3′)-O-(4 Benzoylbenzoyl)adenosine 5′-triphosphate, before evaluating P2X7R protein expression. Finally, by immunohistochemistry and immunofluorescence confocal analysis, we investigated the P2X7R expression in frontal cortex from secondary progressive MS cases. We demonstrated that P2X7R is present and inhibited on peripheral monocytes isolated from MS donors during the acute phase of the disease, moreover it is down-regulated in human monocytes after pro-inflammatory stimulation *in vitro*. P2X7R is instead up-regulated on astrocytes in the parenchyma of frontal cortex from secondary progressive MS patients, concomitantly with monocyte chemoattractant protein-1 chemokine, while totally absent from microglia/macrophages or oligodendrocytes, despite the occurrence of inflammatory conditions. Our results suggest that inhibition of P2X7R on monocytes and up-regulation in astrocytes might contribute to sustain inflammatory mechanisms in MS. By acquiring further knowledge about P2X7R dynamics and identifying P2X7R as a potential marker for the disease, we expect to gain insights into the molecular pathways of MS.

## Introduction

Peripheral and central mechanisms provide insights into the cellular basis of neuroinflammation that leads to severe demyelination and neurodegeneration in multiple sclerosis (MS). During both MS and experimental autoimmune encephalomyelitis (EAE), monocyte-derived macrophages are part of the inflammatory CNS infiltrates and accumulate during the disease concomitantly with active demyelination, while CNS-resident microglia-derived macrophages are inert at disease onset and participate to later phases of the disease. Autoreactive myelin-specific T cells then boost inflammation, demyelination and CNS damage, contributing to neurological deficit, and blood–brain barrier dysfunction (1). In addition, astrocytes appear to have a dual role in MS, depending on the disease status and lesion topography, and contributing in both pathogenic alterations and beneficial repair (2–6). In examining those mechanisms that converge in causing inflammatory demyelination, the analysis of frontal cortex constitutes a convenient experimental platform, because profuse lesions in cerebral cortex constitute a significant proportion of MS pathology, and characterize the evolution from a relapsing/remitting early phase into a secondary progressive MS (SPMS) (7–9).

Extracellular purine/pyrimidine nucleotides and nucleosides binding to several different purinergic receptors are among the most diffuse exogenous signals playing important biological functions in the CNS, being responsible for the cell-to-cell communication under normal and pathological conditions (10–14). A member of the purinergic P2X family of ATP-gated ion channels, the P2X7 receptor (P2X7R) (15) is selectively expressed on cells of the hematopoietic lineage (16–19). Moreover, in the nervous system, P2X7R is present on activated microglia (20–22), astrocytes (23–25), oligodendrocytes (26–28), Schwann cells (29), and neurons (30). Despite its wide expression in many cell types participating to MS, only incomplete information is available regarding P2X7-mediated signaling in the disease. For instance, in optic nerve P2X7R expression is augmented in oligodendrocytes and myelin sheaths in MS and EAE before lesion formation, thus contributing to tissue damage; as a consequence, P2X7R blockade prevents oligodendrocyte excitotoxicity and ameliorates EAE (31). P2X7R immunoreactivity is augmented also in activated microglia/macrophages in spinal cord during MS, and extracellular ATP apparently contributes to MS lesion-associated release of interleukin-1β from microglia/macrophages, *via* P2X7R-dependent induction of cyclooxygenase-2 and downstream pathogenic mediators (21). Mice deficient in P2X7R function are more resistant to EAE than wild-type mice, also showing reduced CNS inflammation, axonal damage, and astrocytes activation (32). Furthermore, pharmacological blockade of the receptor remarkably diminishes astrogliosis in rat EAE and alleviates neurological symptoms (24). On the other hand, it was also reported that P2X7R knockdown causes a more severe EAE and that lymphocyte from P2X7R^−/−^ mice proliferate more vigorously *in vitro*, producing reduced levels of IFN-γ and NO, thus suggesting an important role for this receptor in MS lymphocyte homeostasis (33).

The aim of the present work is to further investigate the role of P2X7R in MS pathogenesis, by analyzing its expression in peripheral blood mononuclear cells (PBMCs) from stable and acute phases of relapsing-remitting MS and in human frontal cortex of SPMS.

## Materials and Methods

### Ethical Statement

Blood samples were obtained following acquisition of the study participants’ informed consent. The protocol was approved by ethic committees of San Camillo Forlanini Hospital. Patients enrolled were diagnosed with relapsing-remitting form of MS according to 2005-revised McDonald’s criteria (34). Frontal cortex tissue was collected postmortem by UK MS Tissue Bank at Imperial College, London, and procedures for retrieval, processing, and storage have gained ethical approval.

### Flow Cytometry and Human Monocytes Separation

Peripheral blood mononuclear cells were isolated by a density gradient centrifugation over a Ficoll-Hypaque (Ficoll-Paque PLUS, GE Healthcare) from 20 ml of freshly venous blood from five healthy donors (HD), five relapsing MS patients (MS acute), and five remitting MS patients (MS stable). Cells were stained with pre-titrated Abs, to evaluate the expression of P2X7R within cluster of differentiation 14 (CD14)-positive cells. Briefly, PBMCs (1 × 10^6^) were incubated with P2X7-extracellular epitope antibody (Alomone Labs, Jerusalem, Israel) for 30 min at 4°C. Cells were washed and stained with goat anti-rabbit Alexa Fluor 488-conjugated antibody (Invitrogen, Life Technologies, Monza, MB, Italy), 30 min at 4°C. Cells were washed and stained with anti-CD14 PE (Dako, Aurogene, Rome, Italy) and Live Dead Fixable Aqua Dead Cell Stain Kit (Invitrogen) for 30 min at 4°C.

Monocytes were isolated from PBMCs of HD by using Magnetic Separation with Negative Selection Columns (Miltenyi Biotec, Calderara di Reno, BO, Italy) according to the product manual. Purified monocytes (6 × 10^6^) were cultured in serum-free RPMI 1640 with L-Glutamine, 50 U/ml penicillin, 50 µg/ml streptomycin in 96-well plates.

FACS analysis was performed with FACS CyAn (Beckman Coulter, Pasadena, CA, USA) and with advanced flow cytometry software FlowJo (Tree Star, Ashaland, OR, USA).

### Quantitative Reverse Transcription Polymerase Chain Reaction (RT-qPCR)

Human and rat monocytes or snap-frozen tissues were homogenized in TRIzol (Life Technologies) and total RNA was extracted following the manufacturer’s instructions. UV spectrophotometric analysis of nucleic acids was performed by Nanodrop spectrophotometer at 260 nm to determine concentration. The 260:280 absorbance ratio was used to assess nucleic acids purity. After DNase treatment (Qiagen, Hilden, Germany), 1 µg of total RNA was subjected to retro-transcription by high-capacity RNA-to-cDNA kit (Applied Biosystem, Life Technologies).

Quantitative polymerase chain reaction was carried out using SYBR green (Applied Biosystem, Life Technologies) incorporation with gene-specific primers (Table 1). Relative gene expression was calculated by ΔΔ*Ct* analysis relative to GAPDH.

**Table 1:** List of primer sequences used in this study.

Primer, F: forward primer, R: reverse primer	Sequence, 5′ to 3′	Type of analysis
Rat P2rx7 F	CTGGTGTCCTGCTGAGGAAG	RT-qPCR
Rat p2rx7 R	CTCGTAGTATAGTTGTGGCCCG	RT-qPCR
Human P2rx7 F	ATACAGTTTCCGTCGCCTTG	RT-qPCR
Human P2rx7 R	AACGGATCCCGAAGACTTTT	RT-qPCR
Rat Il-6 F	GAGGATACCACCCACAACAGACC	RT-qPCR
Rat Il-6 R	CAGTGCATCATCGCTGTTCATACA	RT-qPCR
Rat GAPDH F	GCATCTTCTTGTGCAGTGCC	RT-qPCR
Rat GAPDH R	TACGGCCAAATCCGTTCACA	RT-qPCR
Human GAPDH F	TCTTTTGCGTCGCCAGCCGAG	RT-qPCR
Human GAPDH R	TGACCAGGCGCCCAATACGAC	RT-qPCR

*RT-qPCR, quantitative reverse transcription polymerase chain reaction*.

### EAE Rat Model

Female Lewis rats (~160 g, 6 weeks old) were purchased from Charles River (Como, Italy). Animal procedures were performed according to European Guidelines for animal use in research (86/609/CEE) and requirements of Italian laws (D.L. 116/92), according to protocol no. 112/2014B by Italian Ministry of Health. Efforts were made to minimize animal suffering and the number of animals used.

Female rats were deeply anesthetized and injected in each hind paw with 100 µl of a medium containing 0.15 g/ml guinea pig spinal cord tissue in saline (0.9% NaCl) and complete Freund’s adjuvant (CFA, Sigma-Aldrich, Milan, Italy), 50% vol/vol, to which 5 mg/ml heat-inactivated *Mycobacterium tuberculosis* (Difco H37Ra) were added. CFA-injected rats were used as control of inflammation.

Starting at 5-day postinjection, all animals were daily weighed, assessed for clinical signs of disease, and graded according to the following described criteria: 0 = no clinical signs; 1 = loss of tail tonus; 2 = weakness in one or both hind legs or mild paresis; 3 = severe paresis or paralysis of both hind legs; 4 = severe paralysis of complete lower part of the body; and 5 = death due to aggressive EAE (35).

### Rat and Mouse Monocytes Separation

Female C57BL/6 mice (~25 g, 8 weeks old) were purchased from Charles River (Como, Italy). Animal procedures were performed according to European Guidelines for animal use in research (86/609/CEE) and requirements of Italian laws (D.L. 116/92), according to protocol no. 119/2015PR by Italian Ministry of Health. Efforts were made to minimize animal suffering and the number of animals used.

Female Lewis rats (*n* = 4) and C57BL/6 mice (*n* = 3) were sacrificed by asphyxiation with CO_2_ and spleen excised for monocytes purification. CFA (*n* = 3) and EAE (*n* = 4) rats were sacrificed at 15 days postinjection by asphyxiation with CO_2_. After spleen harvest, single cell suspension was obtained by mechanical tissue dissociation in RCB buffer (NH4Cl 150 mM, NaHCO3 10 mM, and EDTA 1 mM). Cells were plated (4 × 10^6^/ml) in RPMI, 10% fetal bovine serum, 100 U/ml gentamycin, 100 µg/ml streptomycin, and 100 U/ml penicillin. After 2 h, non-adherent cells were removed and medium enriched with 10 ng/ml rat or mouse macrophage colony-stimulating factor (Sigma-Aldrich) (36). After 1 week, cells were used for RT-qPCR, western blotting, and immunofluorescence analysis.

### *In Vitro* Treatments

Human monocytes were stimulated without or with lipopolysaccharide (LPS, Sigma-Aldrich) or 2′(3′)-O-(4-Benzoylbenzoyl)adenosine 5′-triphosphate (BzATP, Sigma-Aldrich) for 4 h (T4) and 24 h (T24) at 37°C in a 5% CO_2_ environment. After treatments, monocytes were incubated with monoclonal antibody as described above, to evaluate P2X7R expression by FACS analysis. Moreover, monocytes from control rats and mice were stimulated *in vitro* with or without LPS or BzATP for 4 h (T4) or 24 h (T24) and P2X7R expression was analyzed by western blotting.

### Human Brain Tissue

Demographic and clinical characteristics of MS cases at the time of tissue collection are reported (Table 2). Frontal cortex tissues are from 13 neuropathological confirmed cases of MS, matched for disease courses (all secondary progressive MS, SPMS) but presenting different ages (range 34–80 years), sex, disease durations (range 6–50 years), and causes of death (see Table 2). Analysis was performed also on samples from four patients who died by non-neurological diseases. Cerebral hemispheres were fixed with 4% paraformaldehyde for 2 weeks, coronally sliced, and blocked. Individual blocks were cryoprotected in 30% sucrose for 1 week, frozen in isopentane, and stored at −80°C until use.

**Table 2:** Summary of patients information.

Case	Age (years)	Sex	Clinical diagnosis	Disease duration (years)	Cause of death	DTPI (h)
MS062	49	F	SPMS	19	Respiratory infection	10
MS073	80	F	SPMS	50	Bronchopneumonia	20
MS074	64	F	SPMS	36	Gastrointestinal bleed/obstruction, aspiration pneumonia	7
MS076	49	F	SPMS	18	Chronic renal failure, heart disease	31
MS079	49	F	SPMS	23	Bronchopneumonia, MS	7
MS088	54	F	SPMS	17	Bronchopneumonia	22
MS105	73	M	SPMS	46	Pneumonia	8
MS114	52	F	SPMS	15	Pneumonia, sepsis, pulmonary embolism	12
MS125	76	F	SPMS	31	MS	13
MS128	78	F	SPMS	50	Small bowel obstruction, pneumonia	22
MS136	40	M	SPMS	9	Respiratory infection	10
MS154	34	F	SPMS	11	Pneumonia	12
MS163	45	F	SPMS	6	Urinary tract infection, MS	28

*Demographic and clinical characteristics of MS cases at the time of tissue collection are reported*.

*DTPI, death-tissue preservation interval; SPMS, secondary progressive multiple sclerosis; MS, multiple sclerosis*.

### Immunohistochemistry

Immunohistochemistry was performed as described (37). Human sections (30–40 µm) were pre-incubated for 10 min with 5% H2O2 in 5% methanol in PBS, and for 24–48 h in PBS-0.3% Triton X-100, 2% normal donkey serum (NDS) at 4°C, with goat anti-P2X7 receptor antibody (1:100, peptide YETNKVTRIQSMNY-C from the *N*-terminus of human P2RX7 corresponding to amino acid residues 13-26, MyBioSource, San Diego, CA, USA). Sections were then incubated with biotinylated donkey anti-goat antibodies (Jackson ImmunoResearch Europe Ltd., Suffolk, UK), followed by avidin–biotin–peroxidase reactions (Vectastain, ABC kit, Vector, Burlingame, CA, USA), using 3,3′-diaminobenzidine (Sigma-Aldrich) as a chromogen. Some sections were counterstained with Luxol fast blue, in order to detect lesion types. Sections were mounted on poly-lysine slides and air dried for 24 h. The histological preparations were examined using an Axioskop 2 light microscope (Zeiss). Images were taken with Neurolucida software (MBF Bioscience, USA).

### Immunofluorescence

Human sections (30–40 µm) were blocked with 10% NDS in 0.3% Triton X-100 in PBS and incubated with primary antisera/antibodies (Table 3) in 0.3% Triton X-100 and 2% NDS in PBS, for 24–48 h at 4°C and processed for double and triple immunofluorescence. The secondary antibodies in 0.3% Triton X-100 and 2% NDS in PBS were Cy3-conjugated donkey anti-goat IgG (1:100, Jackson Immunoresearch, West Grove, PA, USA, red), Cy5-conjugated donkey anti-mouse IgG (1:100, Jackson Immunoresearch, blue), Alexa Fluor^®^ 488-AffiniPure donkey anti-mouse IgG (1:200, Jackson Immunoresearch, green), and Alexa Fluor^®^ 488-AffiniPure donkey anti-rabbit IgG (1:200, Jackson Immunoresearch, green). In the case of biotinylated primary antibody CD14 and Lectin from *Lycopersicon esculentum* (tomato) biotin conjugate (1:200, Sigma-Aldrich), Cy2-streptavidin conjugated secondary antibodies (1:200, Invitrogen) were used.

**Table 3:** Antibodies used in this study.

Antigen	Clone	Epitope (aa)	Target	Dilution	Source
CD45	61D3	^a^	Leukocyte	1:100	Dako
CD14 biotinylated	T29/33	^a^	Monocytes	1:100	eBioscience
CD68	EBM11	^a^	Macrophages/microglia	1:100	Dako
CD68	Polyclonal	100–354	Macrophages/microglia	1:200	Santa Cruz
GFAP	5C10	^a^	Astrocytes	1:500	Novusbio
HLA-DP, DQ, DR (MHC II)	CR3/43	^a^	Macrophages/microglia	1:100	Dako
MBP	2	119–131	Mature oligodendrocytes/myelin	1:100	Chemicon
MCP-1	Polyclonal	62–89	CCL2 chemokine	1:20–1:1,000	ThermoFisher Scientific
P2X7-intracellular receptor, N-terminus	Polyclonal	13–26	P2X7 receptor	1:100	MyBioSource
P2X7-extracellular receptor	Polyclonal	136–152	P2X7 receptor	1:500	Alomone
P2X7-intracellular receptor, C-terminus	Polyclonal	576–595	P2X7 receptor	1:500	Alomone
P2Y12 receptor human	Polyclonal	324–342	Microglia	1:200	Anaspec

*^a^Not specified in the data sheet*.

Rat monocytes maintained in culture for 1 week were fixed with 4% paraformaldehyde for 20 min, permeabilized with 0.1% Triton X-100 for 10 min, blocked in PBS/1% bovine serum albumin, and incubated in PBS/1% bovine serum albumin with anti-cluster of differentiation 68 (CD68, 1:200, Santa Cruz Biotechnology, CA, USA). The secondary antibody was Cy3-conjugated donkey anti-rabbit IgG (1:200, Jackson Immunoresearch). Cells were stained with Höechst 33342 (1:1,000) and covered with coverslip in Fluoromount medium (Sigma-Aldrich) for confocal microscopy.

Quantification of CD68 immunoreactivity was performed with monocytes from rat spleen (from an average of six fields for each animal in each group), using Image J software. Data are expressed as optical density with respect to CFA group.

### Confocal Microscopy

Immunofluorescence analysis was performed by confocal laser scanning microscope (Zeiss, LSM700; Iena, Germany) equipped with four laser lines: 405, 488, 561, and 639 nm. Brightness and contrast were adjusted with Zen software (Zeiss).

### Protein Extraction and Western Blotting

Six different snap-frozen blocks of frontal cortex from three independent SPMS cases, and one block from five different control cases were processed for protein extraction. Detergent-soluble proteins were extracted with Ripa buffer (1% Nonidet P-40, 0.5% sodium deoxycholate, 0.1% SDS in PBS, containing protease inhibitors), using a micropestle. After a short sonication, the homogenates were incubated on ice for 1 h and centrifuged at 13,000 rpm for 10 min at 4°C. To extract detergent-insoluble proteins, the resulting pellet was resuspended in 0.5 M Tris HCl (pH 6.8) containing 2% w/v SDS (same volume as utilized for soluble protein extraction), and incubated at RT for 10 min. Insolubilized material in the pellet (15,000 rpm for 15 min) was discarded. Protein quantification was performed from the supernatants by Bradford colorimetric assay (Biorad, Milan, Italy). Proteins (15 µg) were separated by electrophoresis on 10% SDS-PAGE and transferred to nitrocellulose Hybond-C-extra membranes (Amersham Biosciences, Cologno Monzese, Italy). The filters were pre-wetted in 5% blocking agent in TBS-T (10 mM Tris pH 8, 150 mM NaCl, 0.1% Tween 20) and hybridized overnight with P2X7-extracellular epitope antibody (1:500, peptide KKGWMDPQSKGIQTGRC, corresponding to amino acids 136–152 of mouse P2X7 receptor, Alomone Labs), in the absence or presence of the neutralizing immunogenic peptide used in a 1:1 protein ratio, and with monocyte chemoattractant protein-1 (MCP-1) antibody (1:1,000, ThermoFisher Scientific). The signals were detected with anti-rabbit horseradish peroxidase-conjugated antibody (1: 5,000), and developed by enhanced chemiluminescence (Amersham Biosciences), using Kodak Image Station (KDS IS440CF) and semi-quantitative analysis of which was performed with Image J software.

Total proteins from control (*n* = 4), CFA (*n* = 3), and EAE (*n* = 4) cultured rat monocytes and from control C57BL/6 cultured mouse monocytes (*n* = 3) were obtained after lysis of cells on ice for 1 h in Ripa Buffer and centrifugation at 14,000 rpm for 10 min at 4°C. Protein quantification was performed from the supernatants by Pierce™ BCA Protein Assay Kit (Thermo Scientific, Monza, MB, Italy). Proteins were separated by electrophoresis on 10% SDS-PAGE, transferred to nitrocellulose membranes, and processed for western blotting using P2X7-intracellular epitope antibody [1:500, peptide (C)KIRKEFPKTQGQYSGFKYPY, corresponding to amino acids 576–595 of rat P2X7 receptor, Alomone Labs]. The signal was detected with anti-rabbit horseradish peroxidase-conjugated antibody (1: 2,500), and developed by enhanced chemiluminescence (Amersham Biosciences). The bands of interest were visualized using Kodak Image Station (KDS IS440CF) and semi-quantitative analysis was performed with Image J software.

### Statistical Analysis

Data are presented as mean ± SEM and statistical analysis was determined by ANOVA. Statistical differences between groups were verified by Student’s *t*-test. *p < 0.05, **p < 0.01, and ****p < 0.0001 were considered statistically significant.

## Results

### P2X7 Receptor Is Down-Regulated in Monocytes during Acute MS and EAE

**Figure 1:**
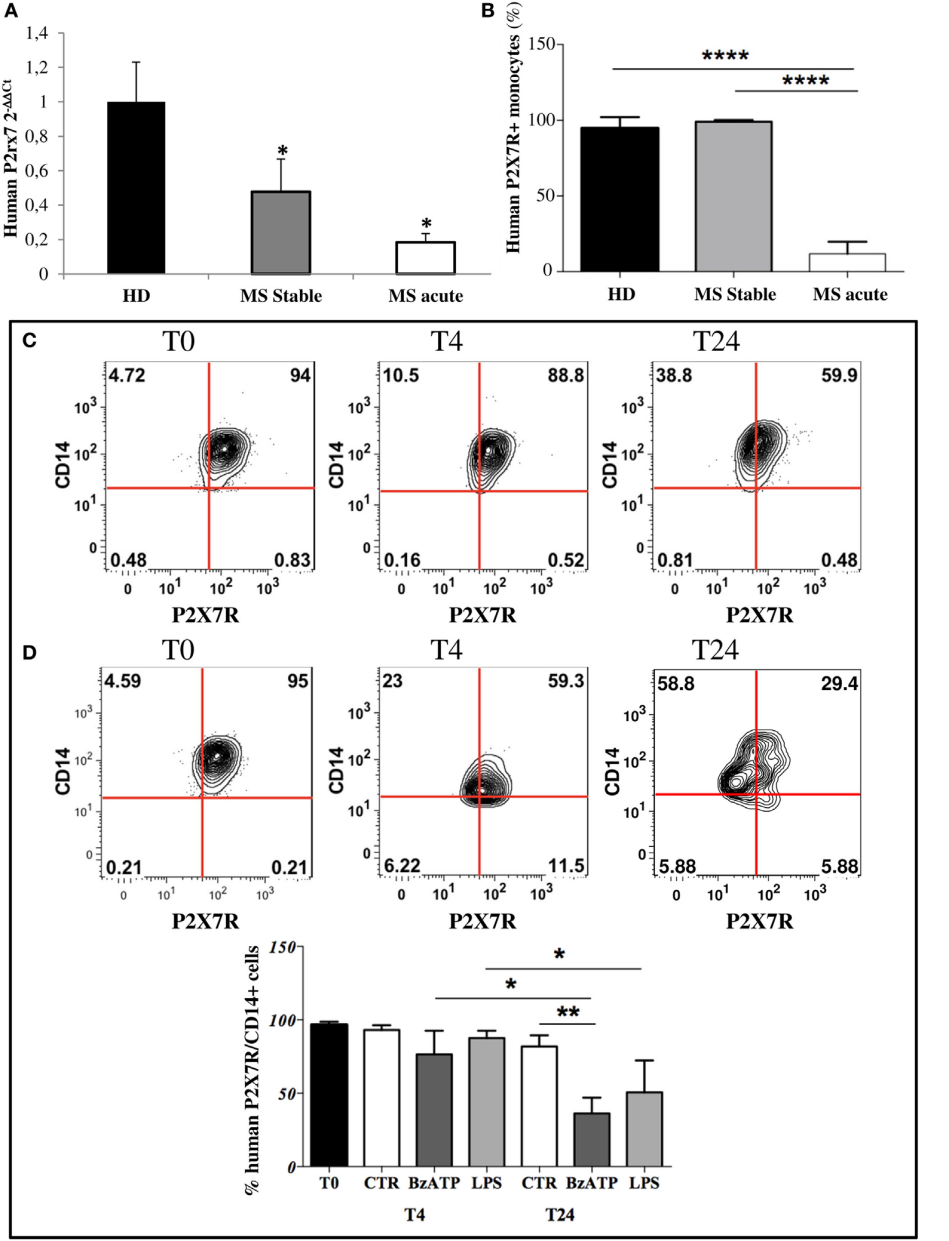
P2X7 receptor (P2X7R) is down-regulated on circulating multiple sclerosis (MS) monocytes and on healthy donors (HD) monocytes after *in vitro* induced inflammation. **(A)** RT-qPCR analysis of P2X7R was performed with freshly isolated monocytes from MS stable (*n* = 8), acute patients (*n* = 8), and HD (*n* = 8). GAPDH was used for normalization. **(B)** Flow cytometry analysis was used to isolate cluster of differentiation 14 (CD14)-positive monocytes and P2X7R-CD14 double-positive cells within freshly isolated peripheral blood mononuclear cells from acute, stable MS patients, and HD. Cumulative data of P2X7R-positive cells within monocytes are reported as % mean ± SEM (*n* = 5). Statistical significance was calculated by ANOVA-Student’s t-test, ****p < 0.0001 and *p < 0.05. Circulating monocytes purified from HD were incubated with inflammatory lipopolysaccharide (250 ng/ml) **(C)** or 2′3′-*O*-(4-benzoyl-benzoyl)adenosine5′-triphosphate (250 µM) **(D)** for 4 and 24 h. Flow cytometry analysis and representative plots of P2X7R expression are shown **(C,D)**, together with cumulative data from three independent experiments are presented **(E)**. Statistical significance was calculated by ANOVA-Student’s t-test *p < 0.05, **p < 0.01.

Given the abundant expression of P2X7R on monocytes (38–40), in this work we firstly confirmed its presence in circulating monocytes from HD and then demonstrated its down-regulation in stable and acute MS patients compared with HD, by RT-qPCR (Figure 1A). Furthermore, FACS analysis confirmed a significant decrease of CD14/P2X7R-positive monocytes only in MS acute patients compared with stable MS and HD conditions (Figure 1B).

Because the rat model of EAE is one among the most commonly used animal model for studying MS pathogenesis, by resembling particularly the acute form of the disease (41), we observed also in EAE monocytes, compared with CFA, a statistically significant decrease of P2X7R mRNA by RT-qPCR (Figure S1A in Supplementary Material), and of protein content by western blot analysis (Figure S1B in Supplementary Material). This occurs in parallel to the increase of the specific monocyte inflammatory marker IL-6 mRNA (Figure S1C in Supplementary Material), and of CD68 protein (Figure S1D in Supplementary Material).

### Inflammatory Stimuli Down-Regulate P2X7 Receptor in Purified Human, Rat, and Mouse Monocytes

In order to mimic an inflammatory insult as it occurs in MS, monocytes from HD were challenged *ex vivo* with LPS or BzATP for 4 and 24 h. A significant decrease of human P2X7R/CD14 bearing monocytes occurred after treatment for 24 h with LPS (Figures 1C,E, ~48% reduction) or BzATP (Figures 1D,E, ~63% reduction), as shown by FACS analysis. Western blot analysis confirmed these results *in vitro* in rat cultured monocytes, showing down-regulation of P2X7R protein after 4 and 24 h of BzATP (Figure S1E in Supplementary Material, ~60 and 90% reduction, respectively) or LPS stimulation for 24 h (Figure S1E in Supplementary Material, ~45% reduction). Similar results were also obtained with mouse purified monocytes challenged *in vitro* with LPS or with BzATP. (Figure S1F in Supplementary Material).

### P2X7 Receptor Is Present on Monocytes in Blood Vessels of SPMS Frontal Cortex

**Figure 2:**
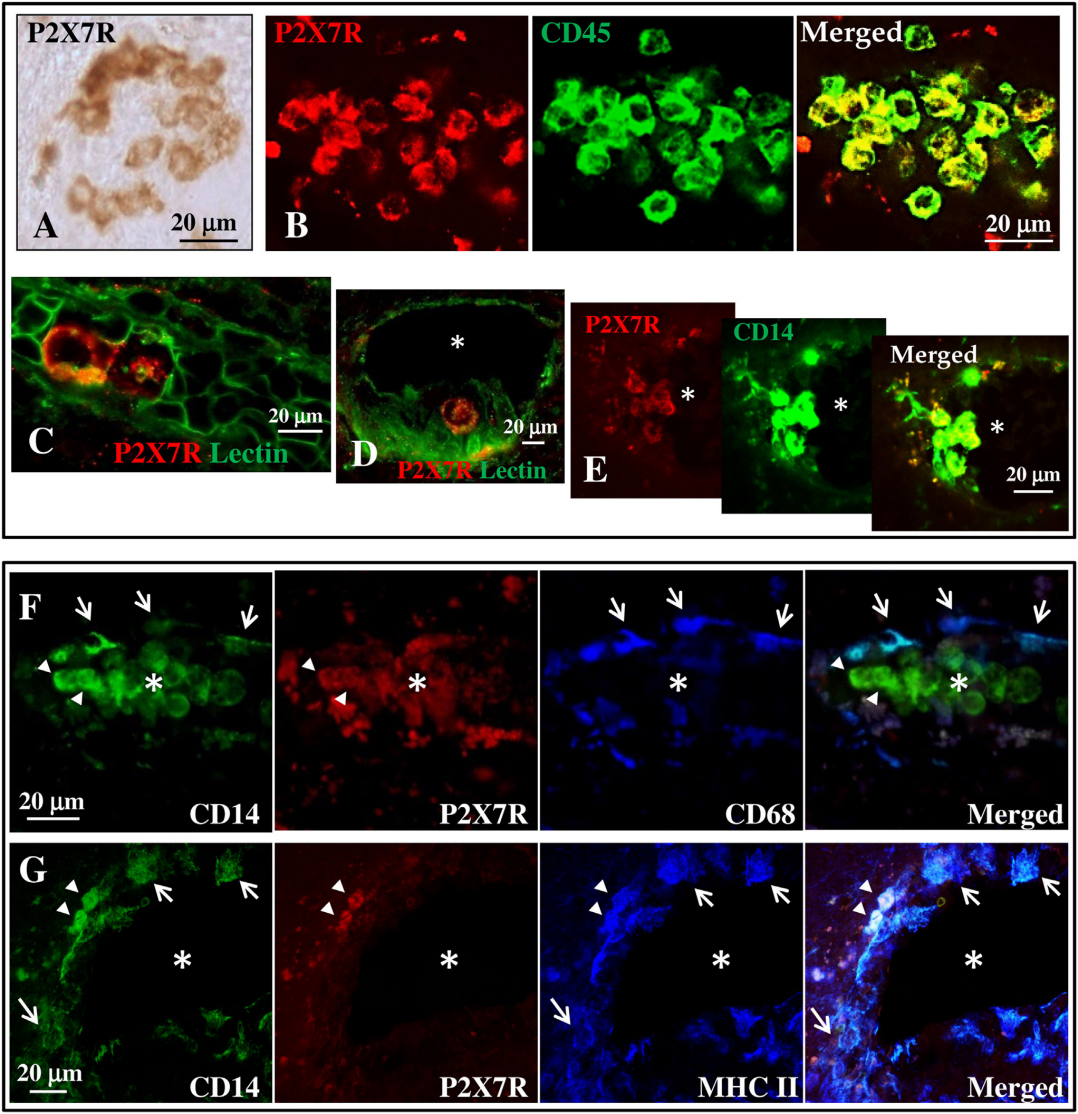
P2X7 receptor (P2X7R) is present on monocytes in blood vessels of secondary progressive multiple sclerosis (SPMS) frontal cortex. **(A)** Immunohistochemistry on sections from human frontal cortex shows roundish P2X7R-positive cells distributed within distinct clusters throughout the entire SPMS tissue. Confocal double immunofluorescence analysis of these clusters exhibits colocalization of P2X7R protein (red) with CD45 leukocyte marker [**(B)**, green]. Staining with Lectin from *Lycopersicon esculentum* for vascular endothelial cells [**(C,D)**, green] clearly shows the presence of P2X7R-positive roundish cells (red) within the lumen of blood vessels (asterisk). Double immunofluorescence of P2X7R-positive clusters shows colocalization of P2X7R (red) with cluster of differentiation 14 (CD14) monocyte/macrophage marker [**(E)**, green]. Confocal triple immunofluorescence analysis is then performed with antibodies for CD14 [**(F,G)**, green], P2X7R [**(F,G)**, red], and microglia/macrophages markers CD68 **(F)** or major histocompatibility complex II [**(G)**, blue]. The asterisk always indicates the lumen of blood vessels, arrows indicate P2X7R-negative cells, and arrowheads P2X7R-positive cells.

By immunohistochemistry and immunofluorescence analysis, we next analyzed the cortical tissue from 13 different cases of SPMS for the presence of P2X7R immunoreactivity and colocalization with specific cellular markers (Tables 2 and 3). In detail, we examined 1–2 different tissue blocks from all cases and inspected 4–10 different slices for each block. The tissue slices were studied in areas either presenting neuronal injury/inflammation, or devoid of visible damage. We observed typical features of cortical demyelination and inflammation in all SPMS cases analyzed. Independent analysis was also performed in cortical tissue from four patients who died by non-neurological diseases (data not shown). In particular, by immunohistochemistry (Figure 2A) the P2X7R immune-positive signal was found to delineate the plasma membrane of roundish cells distributed in distinct clusters in the cortical parenchyma. By immunofluorescence analysis, this roundish P2X7R immune-positive signal (red) was found to colocalize with the CD45 leukocyte marker (Figure 2B, green). By staining with Lectin (green) that specifically delineates endothelial vascular cells, we thus concluded that the roundish P2X7R-positive cluster cells were likely located inside blood vessels (Figures 2C,D) within the SPMS cortical parenchyma. Similar results were confirmed in all cases analyzed (Table 2) and in donors not deceased by neurological conditions (data not shown). By performing double and triple confocal immunofluorescence of these cluster cells with the CD14 monocyte/macrophage marker (Figures 2E–G, green), we demonstrated only partial colocalization with P2X7R immunoreactivity (Figures 2E–G, in red), being the P2X7R signal apparently absent from CD14-positive cells (white arrows) that are proximal to the endothelium of the blood vessels and that are simultaneously positive for CD68 (Figure 2F, blue, white arrows) or for the major histocompatibility complex II (MHC II) (Figure 2G, blue, white arrows) macrophage/microglia markers. In all the sections analyzed, P2X7R is present only on few perivascular double CD14- and MHC II-positive cells (Figure 2G, arrowheads, white signal).

### P2X7 Receptor Is Present on Astrocytes in the Parenchyma of SPMS Frontal Cortex

**Figure 3:**
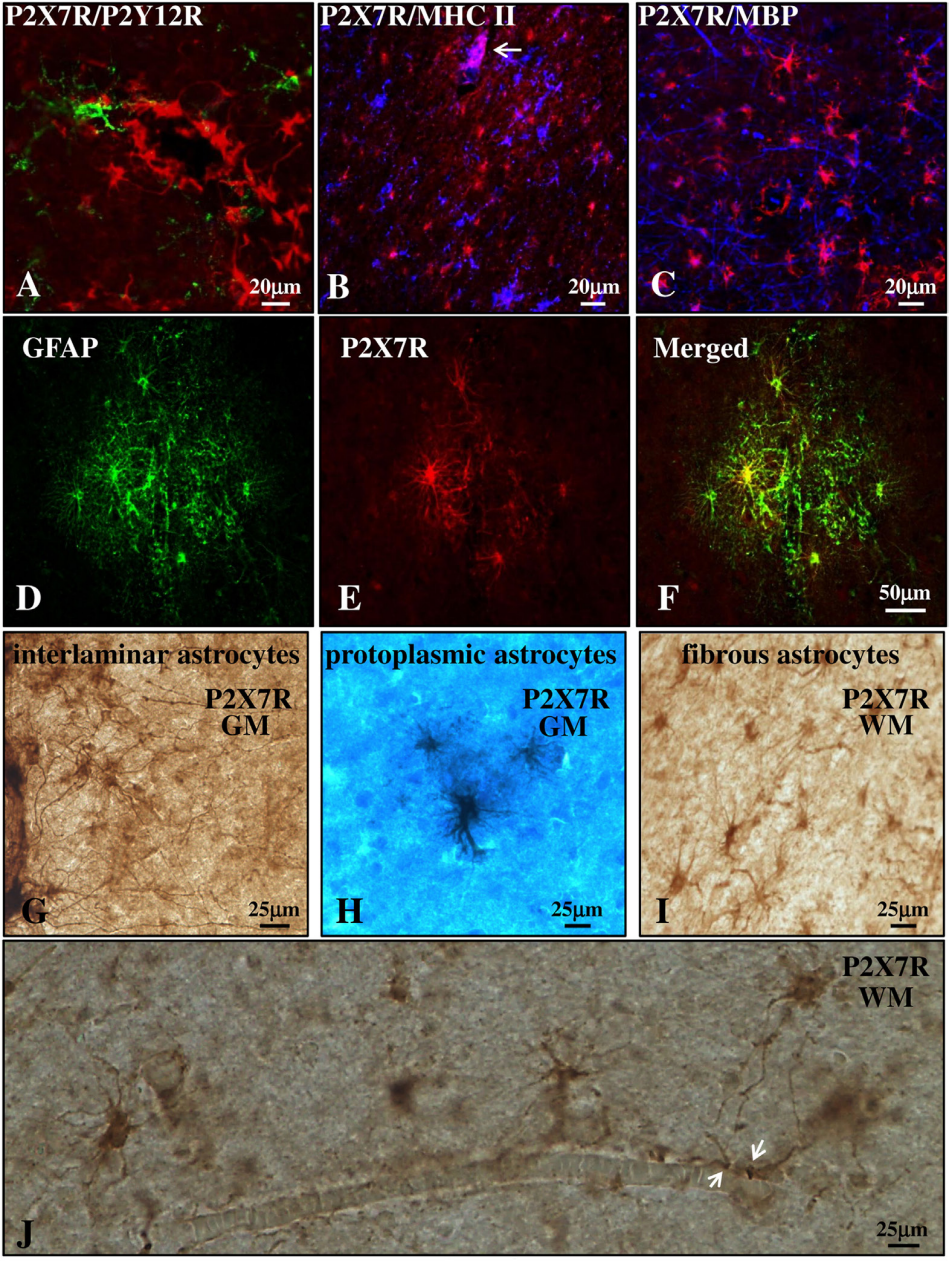
P2X7 receptor (P2X7R) is present on astrocytes in the parenchyma of secondary progressive multiple sclerosis (SPMS) frontal cortex. Confocal analysis of SPMS frontal cortex parenchyma shows absence of colocalization of P2X7R (red) with P2Y12R [**(A)**, green], major histocompatibility complex II (MHC II) [**(B)**, blue], and myelin basic protein [**(C)**, blue], but the presence of merged P2X7R/glial fibrillary acidic protein signals **(D–F)**. P2X7R/MHC II-positive signal is also visible but confined in the lumen of a blood vessel [**(B)**, arrow, pink]. Immunohistochemistry analysis of cortical parenchyma reveals the abundant presence of P2X7R in GM on interlaminar **(G)** and protoplasmic astrocytes **(E)**, and in white matter on fibrous astrocytes **(I,J)**. In **(J)**, astrocytic “vascular feet” are visible adjacent to the blood vessel walls (arrows).

We next investigated the distribution of P2X7R in the cortical parenchyma outside from the blood vessels. Double immunofluorescence confocal analysis indicated the absence of colocalization of P2X7R (red) with P2Y12R (green) or MHC II (blue) (markers, respectively, of quiescent or reactive/active macrophages/microglia, Figures 3A,B) (42, 43), or with myelin basic protein (MBP) (blue, marker of myelin fibers, and oligodendrocyte cell bodies, Figure 3C). The receptor was instead strongly expressed in glial fibrillary acidic protein (GFAP)-positive astrocytes present in both gray (GM, Figures 3D–F) and white matter (WM) (not shown) of both control and MS patients. Moreover, immunohistochemistry studies corroborated the presence of P2X7R in the soma and fibers of interlaminar astrocytes (Figure 3G) present in GM cortical layer I that spread prominent, long, and unbranched processes throughout the layers of the cortex, and moreover in protoplasmic astrocytes (Figure 3H) that are well-organized in GM into non-overlapping spatial domains. In WM, we also observed a strong P2X7R signal in fibrous astrocytes (Figure 3I) that exhibit unbranched cellular processes and that often protrude “vascular feet” (44) that are physically connected to the external capillary walls (Figure 3J).

**Figure 4:**
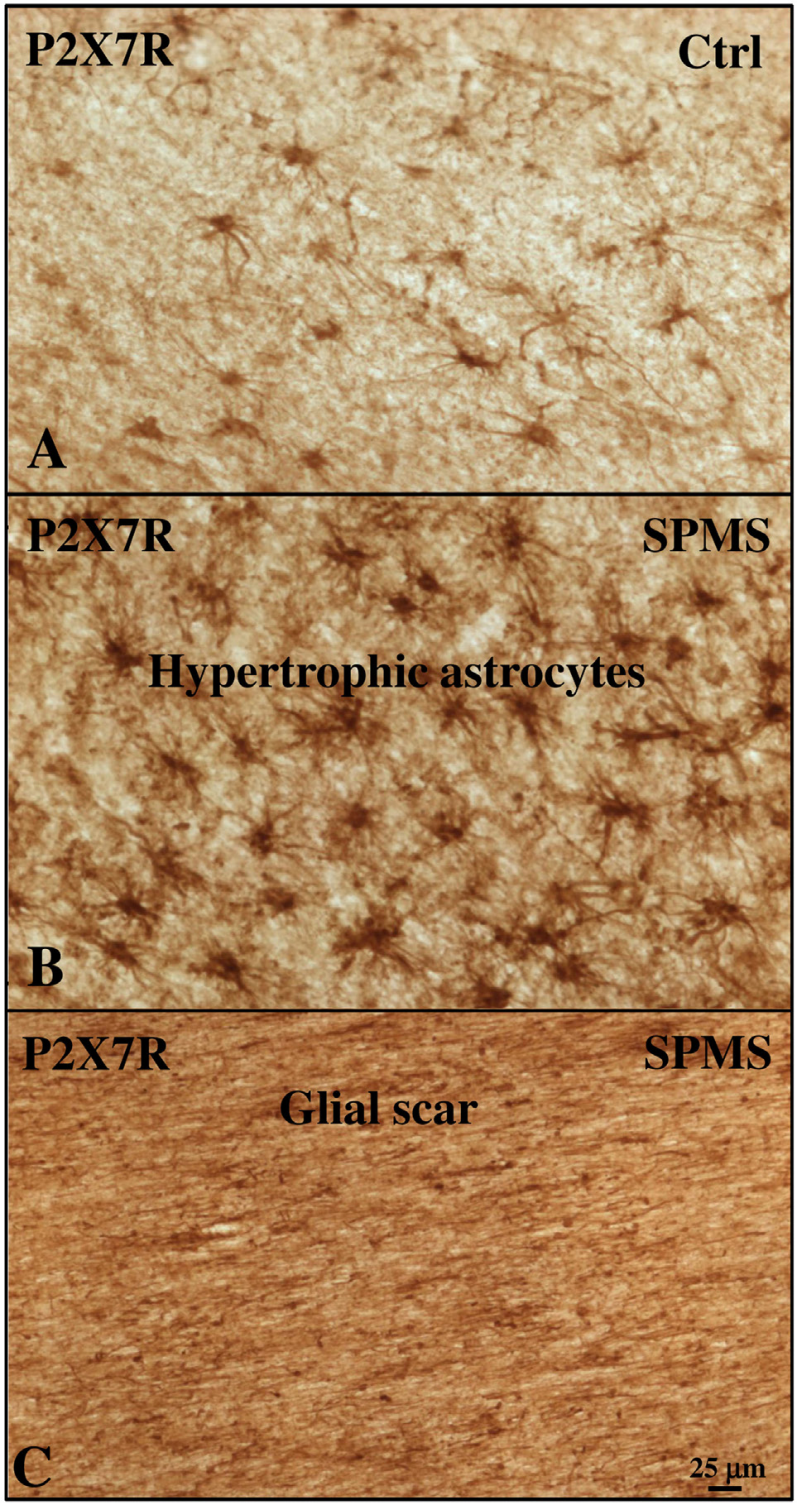
P2X7 receptor (P2X7R) immunoreactivity is increased with astrogliosis in white matter (WM) of secondary progressive multiple sclerosis (SPMS) frontal cortex parenchyma. Immunohistochemistry analysis of two adjacent WM fields from SPMS frontal cortex characterized, respectively, by hypertrophic astrocytes **(B)** and glial scar **(C)** reveals a noteworthy increase in P2X7R-positive astrocytes **(B,C)**, compared with control case **(A)**.

With the aim of further characterizing P2X7R expression and modulation in astrocytes, we acquired images from adjacent immunohistochemical fields within the same cortical sections and compared them with control cases (Figures 4A–C). Respect to control (Figure 4A), we observed a remarkable increase in P2X7R-positive astrocytes in different WM zones of SPMS cortical tissue presenting high levels of astrogliosis (Figure 4B) and glial scar formation (Figure 4C). In detail, in SPMS cortex, P2X7R immunoreactivity distinguished an area (Figure 4B) with strong reactive astrogliosis, intense cellularity, prominent hypertrophy, proliferation, and overlapping of astrocyte processes causing the disruption of distinctive astrocyte domains. Furthermore, the P2X7R signal also identified a zone very rich in parallel and interconnected fibers highlighting a prominent glial scar, where astrocytes displayed densely intersecting processes characterized by intense double P2X7R/GFAP-positive immunoreactive signal (Figure 4C; Figure S2 in Supplementary Material).

### Expression of P2X7 Receptor in Active or Inactive Subpial Lesions of SPMS Frontal Cortex

**Figure 5:**
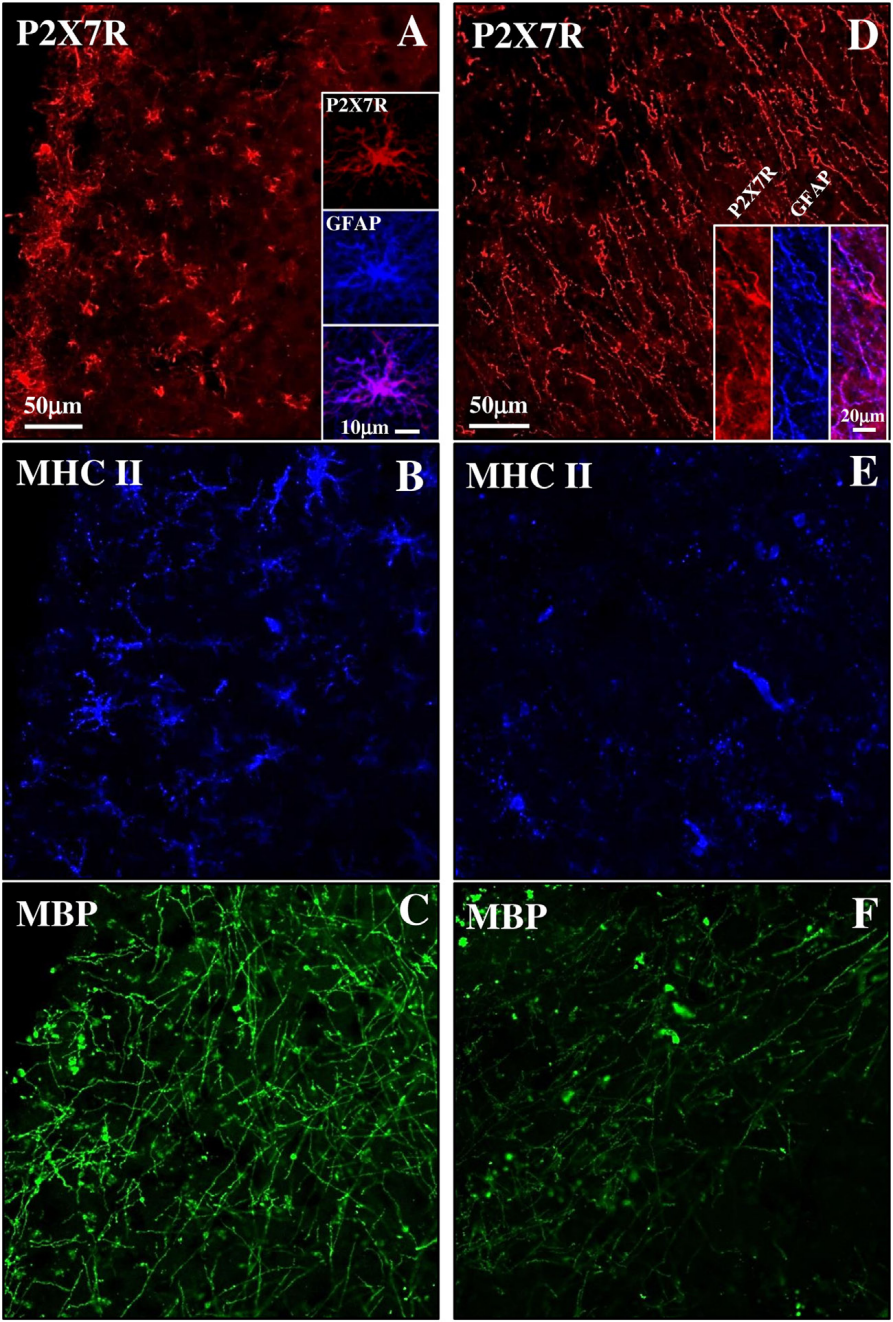
P2X7 receptor (P2X7R) expression in both active and inactive subpial lesions of secondary progressive multiple sclerosis (SPMS) frontal cortex. Confocal triple immunofluorescence analysis performed with antibodies for P2X7R [**(A,D)**, red], major histocompatibility complex II (MHC II) [**(B,E)**, blue], and myelin basic protein (MBP) [**(C,F)**, green] on SPMS frontal cortex sections, shows a chronic active subpial lesion **(A–C)** with abundant glial fibrillary acidic protein (GFAP)/P2X7R-positive signal **(A**, inset**)**, with reactive MHC II-positive monocyets/macrophages/microglia (blue) and with MBP-positive myelin fibers (green). In a chronic inactive lesion **(D–F)**, an intense GFAP/P2X7R glial scar is shown [**(D)**, inset], with decreased MHC II-positive [**(E)**, blue] and MBP-positive [**(F)**, green] immunoreactivities.

Although MS is still widely considered a WM disease, demyelination is also prominent in GM. SPMS phase is characterized by subpial lesions that are highly abundant in progressive stages of MS, closely to the subarachnoid space, involving either part of a cortical gyrus, or often covering adjacent gyri (45). By triple immunofluorescence confocal analysis, we have characterized a subpial lesion with various degrees of inflammatory reaction and demyelination and observed different features of P2X7-positive immunoreactivity. In detail, the presence of profuse reactive MHC II-positive monocytes/macrophages/microglia (blue, Figure 5B) and the permanence of MBP-positive myelin fibers (green, Figure 5C) defined a chronic active lesion characterized by abundant P2X7R-positive signal (red, Figure 5A and insets) that highlighted a zone of intense cellularity and astrogliosis (see merged P2X7R-GFAP signal in the inset). On the other hand, in an area where MHC II (blue, Figure 5E) and MBP (green, Figure 5F) signals both decreased indicating a chronic inactive lesion (46), the P2X7R-positive immunoreactivity (red, Figure 5D) identified a zone very rich in fibers typical of a glial scar (see merged P2X7R-GFAP signal in the inset).

### MS Pathology Alters P2X7 Receptor mRNA and Protein Levels

**Figure 6:**
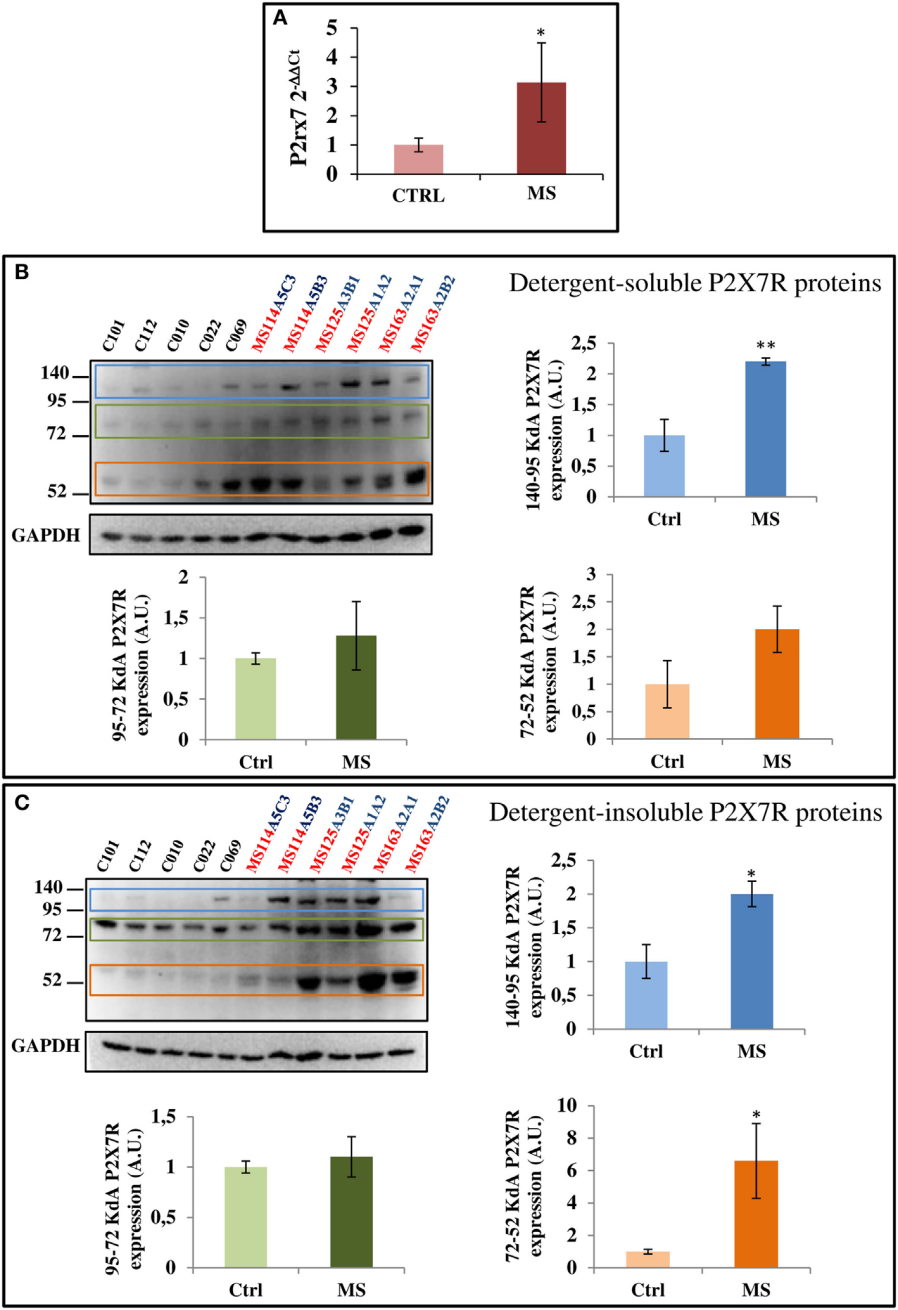
P2X7 receptor (P2X7R) mRNA and protein are augmented in secondary progressive multiple sclerosis. **(A)** Total RNA was extracted from three different snap-frozen blocks from MS patients (cases MS114, MS125, and MS163) and six controls (cases C12–101, C12–112, C13–010, C13–022, C14–069, and C14–053) and the expression of P2X7R mRNA was examined by RT-qPCR. Cortical protein extracts (15 μg/well) from different tissue blocks (A5C3, A5B3, A3B1, A1A2, A2A1, and A2B2) of MS cases MS114, MS125, and MS163 were analyzed by western blotting for the modulation of P2X7R, with respect to control cases (C12–101, C12–112, C13–010, C13–022, and C14–069), in both detergent-soluble **(B)** and -insoluble fractions **(C)**. GAPDH was used for protein normalization. Statistical significance was calculated by Student’s t-test, *p < 0.05, **p < 0.01. Results are shown as mean ± SEM.

To further investigate if SPMS progression modifies the P2X7R content, we analyzed total cell extracts from frontal cortex, by RT-qPCR and western blotting. A statistically significant increase of P2X7R mRNA was observed in SPMS patients, compared with controls (Figure 6A). Similar results were confirmed by immunoblotting with a P2X7R antibody raised against an extracellular epitope of the mouse P2X7R (corresponding to amino acid 136–152). In human frontal cortex tissue, we recognized three specific protein bands with estimated sizes in the ranges 52–72, 72–95, and 95–140 kDa, perhaps corresponding to the different P2X7 isoforms, and that were moreover abolished by the P2X7R neutralizing immunogenic peptide (data not shown). In addition, particularly the 95–140 kDa P2X7R was found significantly up-regulated in both detergent-soluble and -insoluble fractions of SPMS tissue extracts respect to controls, while the 52–72 kDa P2X7R was significantly up-regulated only in the detergent-insoluble fraction of SPMS extracts, with a trend to increase in the detergent-soluble fraction (Figures 6B,C).

### P2X7 Receptor Colocalizes with MCP-1 Chemokine in Human Frontal Cortex

**Figure 7:**
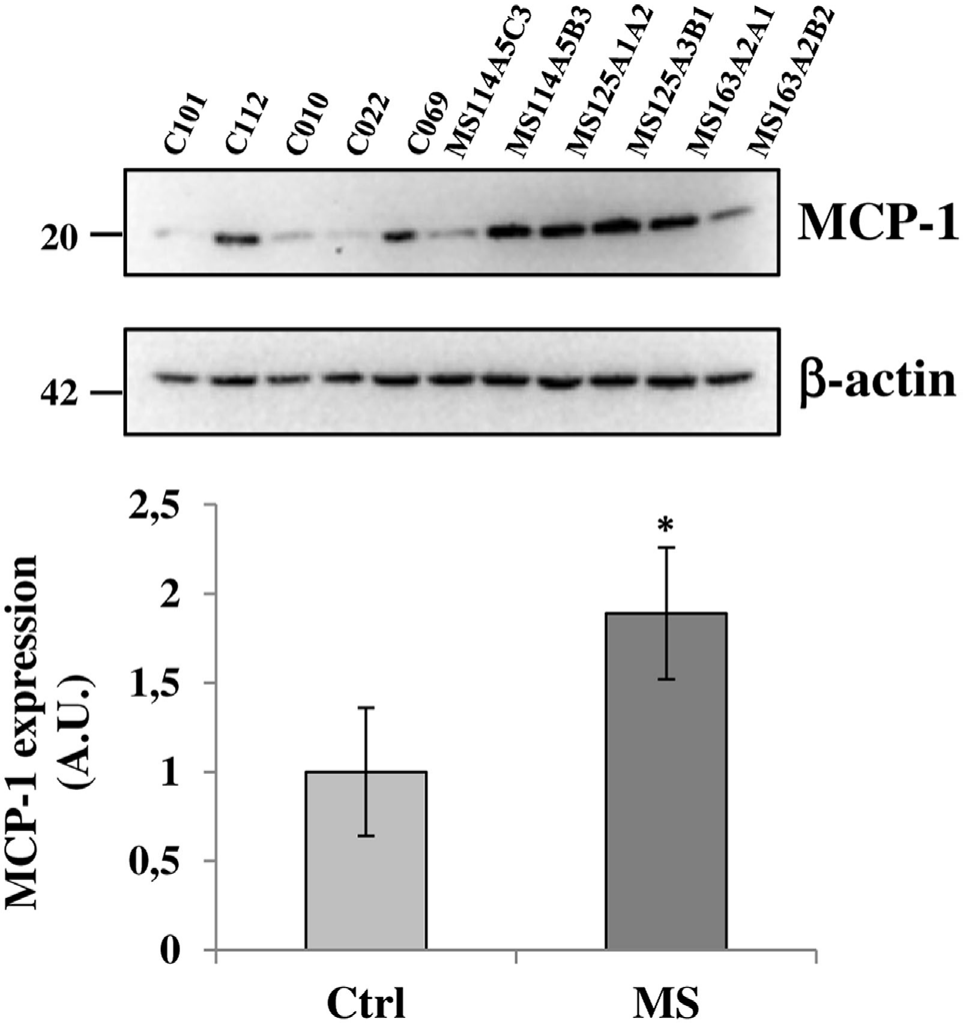
Monocyte chemoattractant protein-1 (MCP-1) chemokine is up-regulated in secondary progressive multiple sclerosis frontal cortex. Equal amount of total protein extracts of cortical tissue (15 μg/well) from different tissue blocks (A5C3, A5B3, A3B1, A1A2, A2A1, and A2B2) of multiple sclerosis (MS) cases MS114, MS125, and MS163 was analyzed by western blotting for the expression of MCP-1 chemokine with respect to control cases (C12–101, C12–112, C13–010, C13–022, and C14–069). β-actin was used for protein normalization. Statistical significance was calculated by Student’s t-test, *p < 0.05. Results are shown as mean ± SEM.

**Figure 8:**
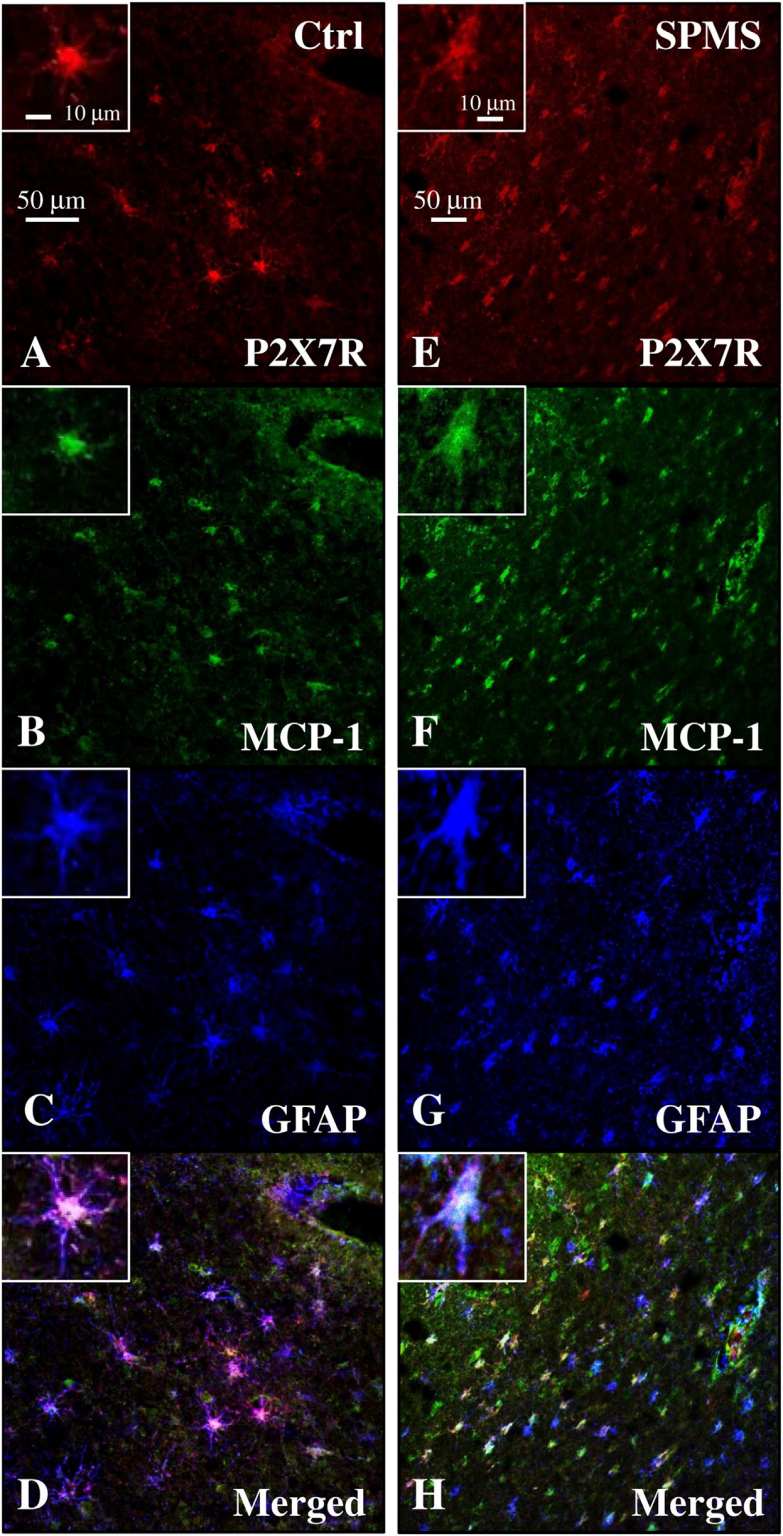
Monocyte chemoattractant protein-1 (MCP-1) chemokine colocalizes with P2X7 receptor (P2X7R) on astrocytes in secondary progressive multiple sclerosis (SPMS) frontal cortex. Triple immunofluorescence confocal analysis performed on sections of frontal cortex from control **(A–D)** and SPMS **(E–H)** with antibodies for P2X7R [**(A,E)**, red], MCP-1 [**(B,F)**, green], and glial fibrillary acidic protein [**(C,G)**, blue] shows colocalization [**(D,H)**, white signal] and strong up-regulation of signals in white matter of SPMS **(E–H)**.

Because the up-regulation of MCP-1 in astrocytes is demonstrated to have an important role in recruiting leukocytes in the CNS during MS (47–49), and the P2X7R agonist BzATP increases MCP-1 expression in astrocytes through P2X7R activation (50), we evaluated the expression of this chemokine in our human SPMS cortical tissue and its potential colocalization with P2X7R on astrocytes. Western blot analysis of protein extracts from control and SPMS patients, demonstrated a strong up-regulation (about twofold increase) of MCP-1 in MS respect to control (Figure 7). Moreover, triple immunofluorescence confocal analysis showed an unambiguous intense colocalization (white signal) among P2X7R (red), MCP-1 (green), and GFAP (blue) proteins, both in control (Figures 8A–D) and SPMS tissues (Figures 8E–H), and moreover a strong up-regulation of both P2X7R and MCP-1 during astrogliosis occurring in WM of SPMS patients (Figures 8E–H).

## Discussion

The adhesion of leukocytes to endothelial cells and their migration into the CNS parenchyma through the blood–brain barrier is a critical step in the development of brain inflammation. Although many types of immune cells are involved in this process during MS progression, activated monocytes are believed to be one of the first phenotypes to reach the brain and initiate neuroinflammation. Because P2X7R is highly expressed in immune cells of the monocyte–macrophage lineage (38), and activation of P2X7R triggers multiple responses affecting the intensity and duration of innate immune and inflammatory reactions in lymphoid leukocytes (51, 52), the aim of this work was to characterize the P2X7R in MS peripheral monocytes and cortical parenchyma. We found that P2X7R protein expression is down-regulated during the acute phase of the disease both in patients and rat EAE monocytes and, moreover, the protein levels of the receptor are reduced in human, rat, and mouse healthy monocytes challenged *in vitro* with pro-inflammatory stimuli. Based on these results, we can hypothesize that high-P2X7R expression might be perhaps deleterious for monocyte survival, and therefore the receptor might be reduced during MS to contribute to initiate and propagate the neuroinflammation. Confirming our hypothesis, overexpression of P2X7R is induced in human monocytes/macrophages infected with high-apoptogenic *M. tuberculosis*, in a suicide-leading track as an attempt to reduce mycobacterial viability (53). Similarly, P2X7R activation with resultant Ca^2+^ overload triggers death also of native mouse monocytes/macrophages (18). Finally, Treg cells with high levels of P2X7R expression are prompted to die (54) when the clearance of excessive toxic ATP is less efficient, as in MS patients with reduced levels of the CD39 ectonucleotidase enzyme (55). Therefore, when high-extracellular concentrations of ATP are released in damaged areas as a result of tissue injury, the down-regulation of P2X7R expression as we demonstrated here, seems an attempt to limit a long-lasting opening of P2X7R channel and massive Ca^2+^ entry, with the final aim of sustaining monocyte survival and, in the case of MS, enhancing pro-inflammatory signals and further damage into the CNS. While this mechanism would apparently diverge from what observed in Behçet’s disease where P2X7R is instead up-regulated in monocytes from patients (56), it is however conserved in pathological conditions other than MS, and in additional cell phenotypes. Reduction of P2X7R expression in PBMCs leading to intracellular calcium dysregulation occurs for instance during ALS (40), a neuroinflammatory/neurodegenerative disease also involving P2X7R (14, 57, 58); P2X7R^−/−^ oligodendrocytes show increased survival in EAE (31), and increased survival of oligodendrocyte precursors occurs also after down-regulation of P2X7R during hypoxia ischemia (59).

We have next looked at P2X7R expressed in CNS tissue, and found that the P2X7R is localized on CD45/CD14-positive monocytes that are visible in the lumen of blood vessels of the cortical parenchyma. Remarkably, the receptor was progressively lost on both CD14/CD68- or CD14/MHC-II-positive cells neighboring the endothelium of the blood vessels and perhaps entering into the CNS, thus corroborating the hypothesis that inflammatory stimuli in the peripheral tissue might trigger a secondary autocrine/paracrine down-regulation of P2X7R expression, with the final aim of boosting and propagating the neuroinflammation into the CNS.

Although these results validate the renowned importance of purinergic P2X7R-dependent signaling in neuroinflammatory conditions, the impact of this receptor in the pathogenesis and clinical aspects of MS is still to be defined. In order to clarify how P2X7R down-regulation in monocytes might correlate to inflammatory lesions and disease progression, we investigated its expression also in autoptic cortical tissue from SPMS donors. In contrast to optic nerve from rat EAE (31) and MS patients (32), in SPMS frontal cortex we found P2X7R absent from myelin fibers and oligodendrocyte cell bodies. Differently from MS spinal cord (21), in SPMS frontal cortex we did not detect P2X7R expression on resting and activated microglia. In contrast to other MS cerebral areas, in SPMS frontal cortex we found P2X7R also absent from neurons, although we confirmed its presence in astrocytes (23). In particular, abundant P2X7R immunoreactivity was found localized on interlaminar and protoplasmic astrocytes of gray matter, and on fibrous astrocytes of white matter. Noticeably, P2X7R-positive astrocytes augmented in cortical tissue of SPMS patients in proximity of lesions. By further extending previous results, these observations thus indicate that P2X7R localization in the CNS and modulation in MS is strictly tissue- and specie-specific.

Being P2X7R *per se* involved in several and sometimes opposite functions (15), its presence on astrocytes playing a dual role in MS by either promoting inflammation and impeding tissue repair, or protecting healthy tissue from adjacent zones of strong inflammation (60), can designate its potential role in several distinct actions. Indeed, we might hypothesize that in astrocytes of SPMS frontal cortex, the up-regulation of P2X7R might contribute to build the parenchyma micro-architecture, being the receptor expressed by interlaminar, protoplasmic, and fibrous astrocytes (61–63). P2X7R could also regulate extracellular K+ homeostasis and participate to the removal of excess glutamate, by directly modulating K+ efflux (64, 65). Moreover, increased P2X7R might influence the connectivity of neuronal circuits, being the receptor known to be involved in the control of myelination (29), or provide metabolic support to neurons, by regulating the lipid metabolism pathway (66). Finally, P2X7R might participate to preserve the blood–brain barrier (60), since we demonstrated here its expression in astrocytes protruding their vascular feet on external capillary walls. However, the specific role of P2X7R up-regulation on SPMS astrocytes might be regulated by several features, including the specific disease stage, the presence of active or inactive lesions characterized, respectively, by P2X7R-positive astrogliosis or P2X7R-positive glial scar, the interaction with various cell types, such as for instance the endothelial cells. Extracellular soluble factors might also influence the diverse cellular reactivity to P2X7R activation during MS. For instance, extracellular ATP is known to induce the secretion from astrocytes of MCP-1 (50), a crucial chemokine up-regulated after CNS trauma (67), and known to have an important role in engaging monocyte-rich infiltrates into the CNS during MS (47–49). In addition, BzATP increases *in vitro* MCP-1 levels in cultured astrocytes directly through P2X7R activation (50). On this regard, the increase of MCP-1 protein that we have demonstrated in SPMS cortical extracts might likely be related to the up-regulation of P2X7R in astrocytes. Supporting this possibility, we have also demonstrated that MCP-1 is induced in cortical MS protein extracts and indeed colocalizes with P2X7R on astrocytes in SPMS frontal cortex.

**Figure 9:**
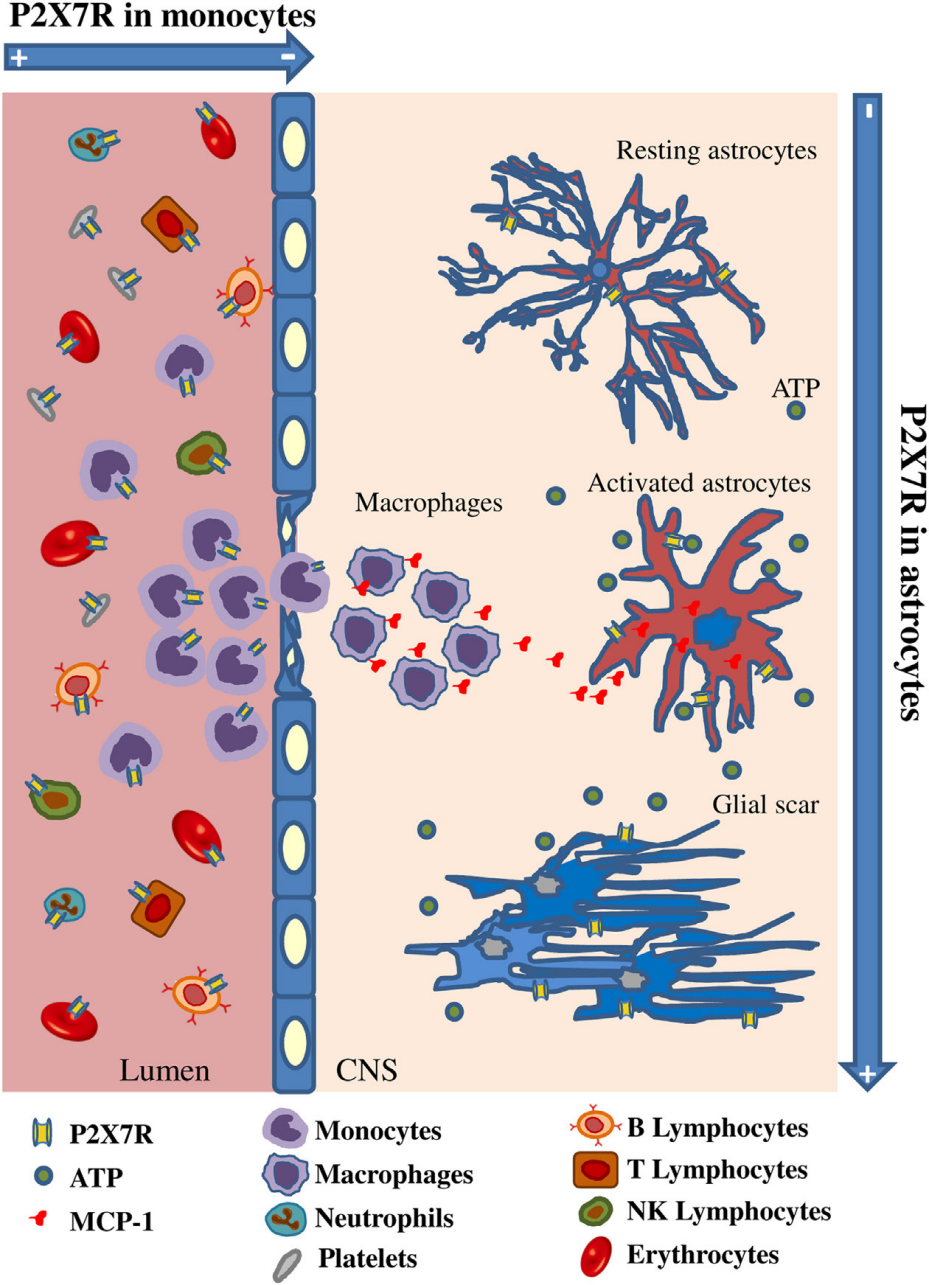
Spatiotemporal profile of P2X7 receptor (P2X7R) expression in multiple sclerosis (MS). The cartoon describes that P2X7R is down-regulated in monocytes during their activation and extravasation from blood vessel to MS cerebral cortex, while the receptor and the monocyte chemoattractant protein-1 chemokine are up-regulated on MS astrocytes concurrently with increased glial fibrillary acidic protein signal and glial scar formation.

By considering our results in the context of previous knowledge, we can therefore formulate the following hypothesis: extracellular ATP is increased in CNS tissue as an alarm signal due to progressive homeostasis loss during MS; astrocytes up-regulate P2X7R and MCP-1; this last functions as attractant for peripheral monocytes which in turn down-regulate P2X7R to guarantee their survival and invasion into the CNS tissue, thus contributing to the detrimental effects of neuroinflammation (Figure 9). Further work will verify our hypothesis and the time-cause correlation of these events. By gaining insights into P2X7R dynamics and signaling, we expect to contribute to further discern some molecular aspects of MS.

## Ethics Statement

Blood samples were obtained following acquisition of the study participants’ informed consent. The protocol was approved by ethic committees of San Camillo Forlanini Hospital. Patients enrolled were diagnosed with relapsing-remitting form of MS according to 2005-revised McDonald’s criteria (34). Prefrontal cortex tissue was collected postmortem by UK MS Tissue Bank at Imperial College, London, and procedures for retrieval, processing, and storage have gained ethical approval. Animal procedures were performed according to European Guidelines for animal use in research (86/609/CEE) and requirements of Italian laws (D.L. 116/92), according to protocols no. 112/2014B and no. 119/2015PR by Italian Ministry of Health. Efforts were made to minimize animal suffering and the number of animals used.

## Author Contributions

SAm and CV: study concept and design. SAm, CP, PF, EP, SAp, CM, FLP, LB, and CV: data acquisition and analysis. SR and CG: collection and selection of blood samples and/or clinical data. SAm, CP, EP, and CV: manuscript and/or figures drafting. SL participated in interpreting results and critical reading of the manuscript. LB supervised flow cytometry analysis and contributed with immunology expertise. All authors edited and approved the final version of the manuscript.

## Conflict of Interest Statement

The authors declare that the study was performed in the absence of any commercial or financial relationships that could be interpreted as a potential conflict of interest.

## Supplementary Material

Figure S1 P2X7R is downregulated on spleen monocytes from rat EAE, and on rat and mouse spleen monocytes after pro-inflammatory induction with LPS and BzATP. CFA and EAE purified rat monocytes were subjected to RT-qPCR **(A)** and western blot analysis **(B)** for evaluation of P2X7R expression. Data in **(A,B)** represent means ± SEM (*n* = 4 in EAE and *n* = 3 in CFA). RT-qPCR **(C)** and immunofluorescence analysis **(D)**, respectively for IL-6 mRNA and CD68 protein is shown. Data represent means ± SEM (*n* = 3 in EAE, and *n* = 3 in CFA). Statistical significance was calculated by Student’s *t*-test, **p* < 0.05 compared to CFA. Western blot analysis of P2X7R expression after *in vitro* administration of pro-inflammatory BzATP (250 μM) or LPS (100 ng/ml) for 4 and 24 h to purified rat **(E)** and mouse **(F)** monocytes. Equal amount of total protein (3–6 μg/well) was analyzed in each sample and GAPDH was used for protein normalization. Results are shown as means ± SEM, *n* = 4 for rat and *n* = 3 for mouse. Statistical significance was calculated by Student’s *t*-test, **p* < 0.05, ***p* < 0.01 compared to T0.

Figure S2 P2X7R/GFAP-positive astrocytes characterize a glial scar in WM chronic lesion of SPMS frontal cortex. Confocal double immunofluorescence analysis performed with antibodies for P2X7R (red) and GFAP (blue) on SPMS frontal cortex sections, shows a chronic lesion in WM characterized by the presence of a glial scar with abundant P2X7R/GFAP double-positive fibers.

## Abbreviations

BzATP, 2′(3′)-O-(4-Benzoylbenzoyl)adenosine 5′-triphosphate; CD, cluster of differentiation; CFA, complete Freund’s adjuvant; EAE, experimental autoimmune encephalomyelitis; GFAP, glial fibrillary acidic protein; HD, healthy donors; LPS, lipopolysaccharide; MBP, myelin basic protein; MHC II, major histocompatibility complex II; MS, multiple sclerosis; NDS, normal donkey serum; PBMCs, peripheral blood mononuclear cells; P2X7R, P2X7 receptor; RT-qPCR, quantitative reverse transcription polymerase chain reaction; SPMS, secondary progressive MS.
