# Supplementary figures and images for: Modulation of P2X7 Receptor during Inflammation in Multiple Sclerosis
Source: Front Immunol. 2017 Nov 15;8:1529. doi: 10.3389/fimmu.2017.01529 (PMC5694754; doi:10.3389/fimmu.2017.01529)

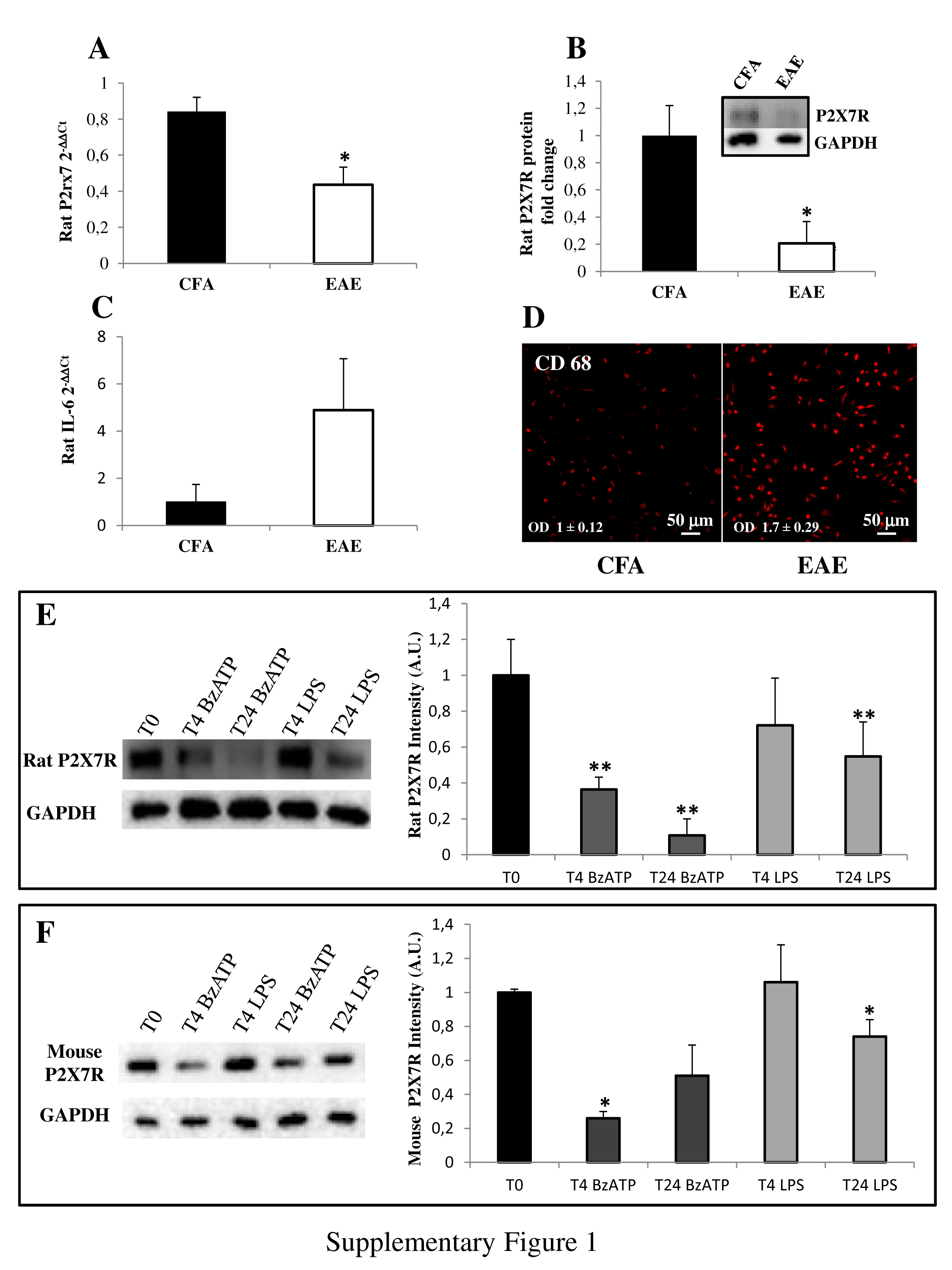

Supplement: Figure S1 — P2X7R is downregulated on spleen monocytes from rat EAE, and on rat and mouse spleen monocytes after pro-inflammatory induction with LPS and BzATP. CFA and EAE purified rat monocytes were subjected to RT-qPCR (A) and western blot analysis (B) for evaluation of P2X7R expression. Data in (A,B) represent means ± SEM (n = 4 in EAE and n = 3 in CFA). RT-qPCR (C) and immunofluorescence analysis (D), respectively for IL-6 mRNA and CD68 protein is shown. Data represent means ± SEM (n = 3 in EAE, and n = 3 in CFA). Statistical significance was calculated by Student’s t-test, *p < 0.05 compared to CFA. Western blot analysis of P2X7R expression after in vitro administration of pro-inflammatory BzATP (250 μM) or LPS (100 ng/ml) for 4 and 24 h to purified rat (E) and mouse (F) monocytes. Equal amount of total protein (3–6 μg/well) was analyzed in each sample and GAPDH was used for protein normalization. Results are shown as means ± SEM, n = 4 for rat and n = 3 for mouse. Statistical significance was calculated by Student’s t-test, *p < 0.05, **p < 0.01 compared to T0. [file Data_Sheet_1.zip › Supplementary figure 1.tif]

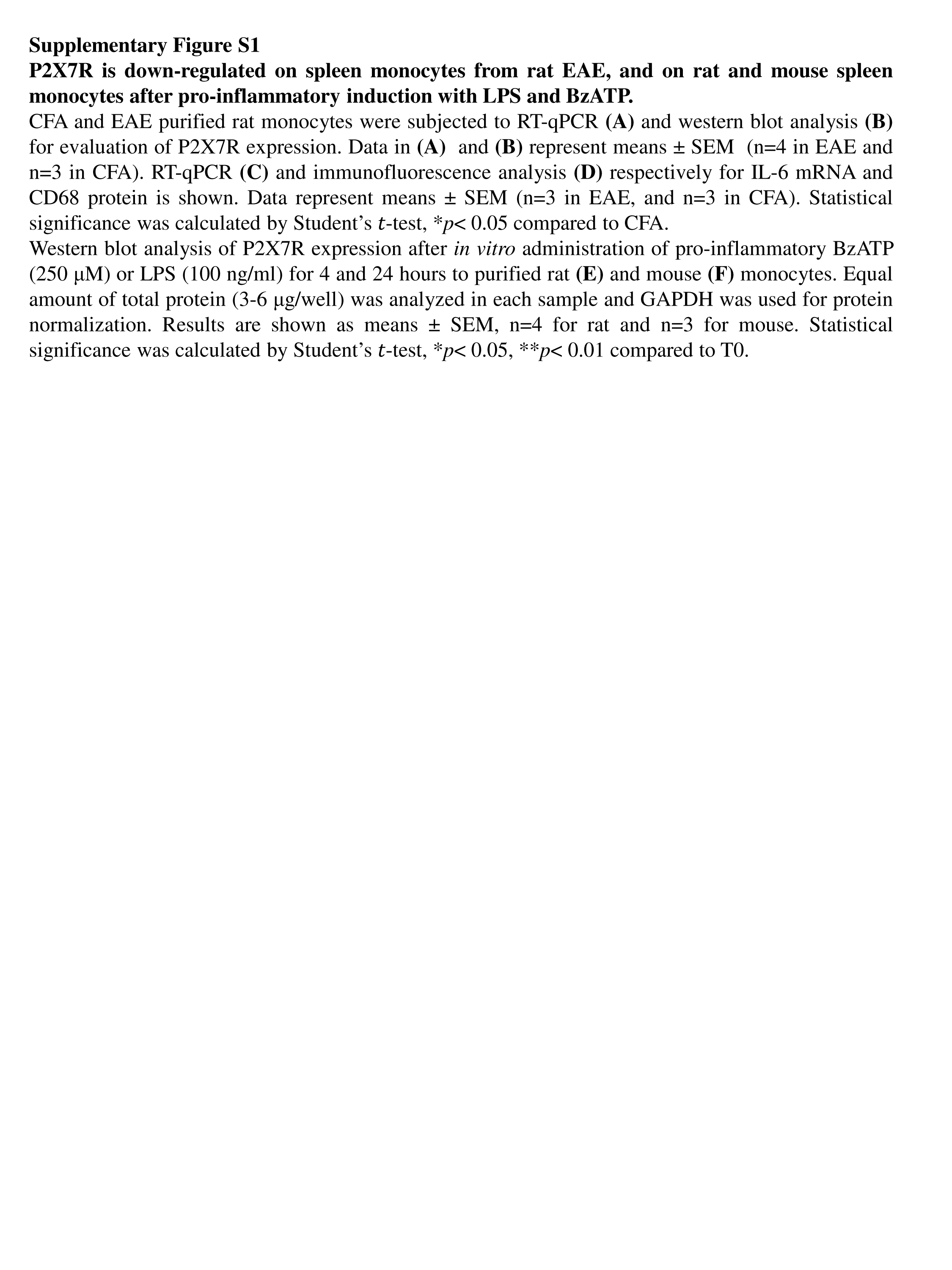

Supplement: Figure S1 — P2X7R is downregulated on spleen monocytes from rat EAE, and on rat and mouse spleen monocytes after pro-inflammatory induction with LPS and BzATP. CFA and EAE purified rat monocytes were subjected to RT-qPCR (A) and western blot analysis (B) for evaluation of P2X7R expression. Data in (A,B) represent means ± SEM (n = 4 in EAE and n = 3 in CFA). RT-qPCR (C) and immunofluorescence analysis (D), respectively for IL-6 mRNA and CD68 protein is shown. Data represent means ± SEM (n = 3 in EAE, and n = 3 in CFA). Statistical significance was calculated by Student’s t-test, *p < 0.05 compared to CFA. Western blot analysis of P2X7R expression after in vitro administration of pro-inflammatory BzATP (250 μM) or LPS (100 ng/ml) for 4 and 24 h to purified rat (E) and mouse (F) monocytes. Equal amount of total protein (3–6 μg/well) was analyzed in each sample and GAPDH was used for protein normalization. Results are shown as means ± SEM, n = 4 for rat and n = 3 for mouse. Statistical significance was calculated by Student’s t-test, *p < 0.05, **p < 0.01 compared to T0. [file Data_Sheet_1.zip › Supplementary figure 1 legend.tif]

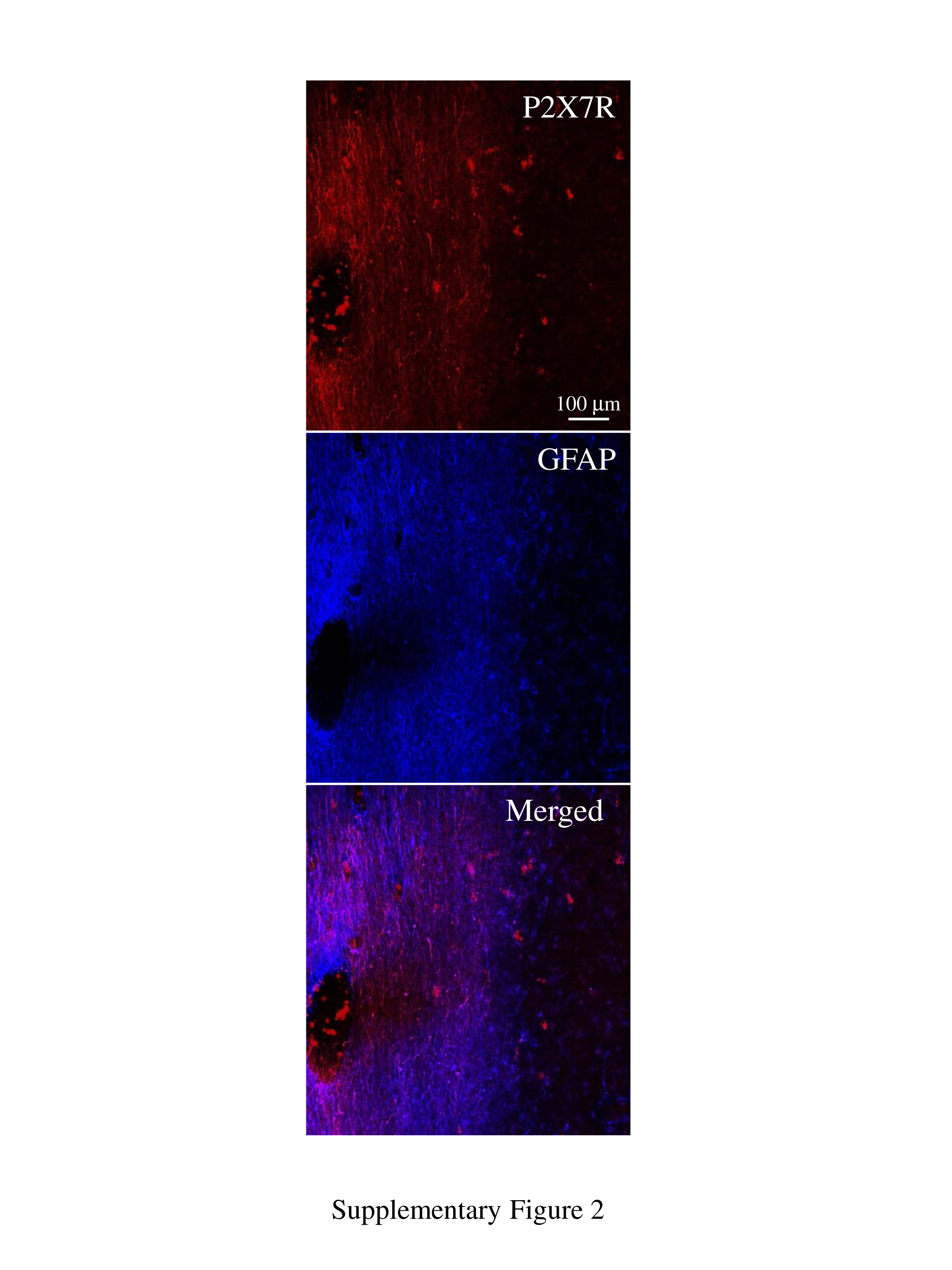

Supplement: Figure S2 — P2X7R/GFAP-positive astrocytes characterize a glial scar in WM chronic lesion of SPMS frontal cortex. Confocal double immunofluorescence analysis performed with antibodies for P2X7R (red) and GFAP (blue) on SPMS frontal cortex sections, shows a chronic lesion in WM characterized by the presence of a glial scar with abundant P2X7R/GFAP double-positive fibers. [file Data_Sheet_2.zip › Supplementary figure 2.tif]

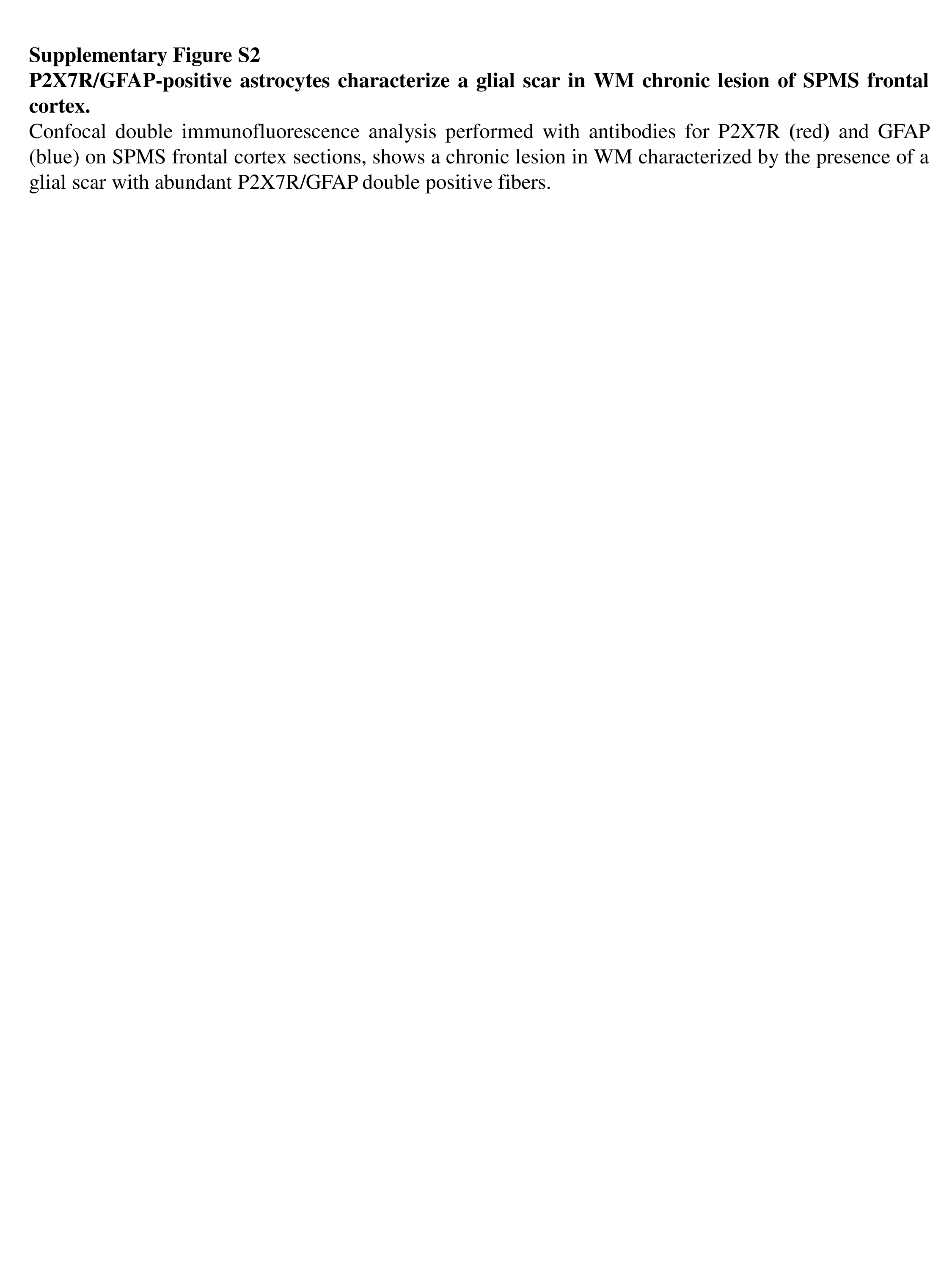

Supplement: Figure S2 — P2X7R/GFAP-positive astrocytes characterize a glial scar in WM chronic lesion of SPMS frontal cortex. Confocal double immunofluorescence analysis performed with antibodies for P2X7R (red) and GFAP (blue) on SPMS frontal cortex sections, shows a chronic lesion in WM characterized by the presence of a glial scar with abundant P2X7R/GFAP double-positive fibers. [file Data_Sheet_2.zip › Supplementary figure 2 legend.tif]
